# Risk Factors Associated With SARS-CoV-2 Infections, Hospitalization, and Mortality Among US Nursing Home Residents

**DOI:** 10.1001/jamanetworkopen.2021.6315

**Published:** 2021-03-31

**Authors:** Hemalkumar B. Mehta, Shuang Li, James S. Goodwin

**Affiliations:** 1 Center for Drug Safety and Effectiveness, Johns Hopkins Bloomberg School of Public Health, Baltimore, Maryland; 2 Department of Epidemiology, Johns Hopkins Bloomberg School of Public Health, Baltimore, Maryland; 3 Sealy Center on Aging, Department of Internal Medicine, The University of Texas Medical Branch, Galveston

## Abstract

**Importance:**

Nursing home residents account for approximately 40% of deaths from SARS-CoV-2.

**Objective:**

To identify risk factors for SARS-CoV-2 incidence, hospitalization, and mortality among nursing home residents in the US.

**Design, Setting, and Participants:**

This retrospective longitudinal cohort study was conducted in long-stay residents aged 65 years or older with fee-for-service Medicare residing in 15 038 US nursing homes from April 1, 2020, to September 30, 2020. Data were analyzed from November 22, 2020, to February 10, 2021.

**Main Outcomes and Measures:**

The main outcome was risk of diagnosis with SARS-CoV-2 (per *International Statistical Classification of Diseases, Tenth Revision, Clinical Modification* [*ICD-10-CM*] codes) by September 30 and hospitalization or death within 30 days after diagnosis. Three-level (resident, facility, and county) logistic regression models and competing risk models conditioned on nursing home facility were used to determine association of patient characteristics with outcomes.

**Results:**

Among 482 323 long-stay residents included, the mean (SD) age was 82.7 (9.2) years, with 326 861 (67.8%) women, and 383 838 residents (79.6%) identifying as White. Among 137 119 residents (28.4%) diagnosed with SARS-CoV-2 during follow up, 29 204 residents (21.3%) were hospitalized, and 26 384 residents (19.2%) died within 30 days. Nursing homes explained 37.2% of the variation in risk of infection, while county explained 23.4%. Risk of infection increased with increasing body mass index (BMI; calculated as weight in kilograms divided by height in meters squared) (eg, BMI>45 vs BMI 18.5-25: adjusted hazard ratio [aHR], 1.19; 95% CI, 1.15-1.24) but varied little by other resident characteristics. Risk of hospitalization after SARS-CoV-2 increased with increasing BMI (eg, BMI>45 vs BMI 18.5-25: aHR, 1.40; 95% CI, 1.28-1.52); male sex (aHR, 1.32; 95% CI, 1.29-1.35); Black (aHR, 1.28; 95% CI, 1.24-1.32), Hispanic (aHR, 1.20; 95% CI, 1.15-1.26), or Asian (aHR, 1.46; 95% CI, 1.36-1.57) race/ethnicity; impaired functional status (eg, severely impaired vs not impaired: aHR, 1.15; 95% CI, 1.10-1.22); and increasing comorbidities, such as renal disease (aHR, 1.21; 95% CI, 1.18-1.24) and diabetes (aHR, 1.16; 95% CI, 1.13-1.18). Risk of mortality increased with age (eg, age >90 years vs 65-70 years: aHR, 2.55; 95% CI, 2.44-2.67), impaired cognition (eg, severely impaired vs not impaired: aHR, 1.79; 95% CI, 1.71-1.86), and functional impairment (eg, severely impaired vs not impaired: aHR, 1.94; 1.83-2.05).

**Conclusions and Relevance:**

These findings suggest that among long-stay nursing home residents, risk of SARS-CoV-2 infection was associated with county and facility of residence, while risk of hospitalization and death after SARS-CoV-2 infection was associated with facility and individual resident characteristics. For many resident characteristics, there were substantial differences in risk of hospitalization vs mortality. This may represent resident preferences, triaging decisions, or inadequate recognition of risk of death.

## Key Points

**Question:**

What risk factors are associated with SARS-CoV-2 infections, hospitalization, and mortality among nursing home residents?

**Findings:**

In this cohort study among 482 323 long-stay residents, risk of SARS-CoV-2 infections were associated with geographic area and the specific facility, not by characteristics of the residents. Among residents diagnosed with SARS-CoV-2 infections, the risk of hospitalization associated with individual resident characteristics differed from the risk of death.

**Meaning:**

These findings suggest that decisions on hospitalization of nursing home residents with SARS-CoV-2 were inconsistently associated with risk of death.

## Teaser

This cohort study examined risk factors associated with SARS-CoV-2 infection incidence, hospitalization, and death among nursing home residents in the US.

## Introduction

While 5% of US SARS-CoV-2 infections have occurred in nursing home residents, they account for almost 40% of deaths.^1,2,3^ The case fatality rates are 5 times higher in long-stay nursing home residents than the national mean.^2^

Large cohort studies conducted in community-dwelling adults have identified important risk factors for SARS-CoV-2–related hospitalization and deaths, such as advanced age, male sex, and comorbidities.^4,5,6^ Nursing home residents typically are very old and frail, have more comorbidities and cognitive dysfunction, and are dependent in activities of daily living. Therefore, risk factors for SARS-CoV-2 outcomes may differ for nursing home residents.

Ecological studies conducted at the nursing home level have explored the role of resident and nursing home characteristics associated with SARS-CoV-2 outcomes.^7,8,9,10^ Mixed evidence exists that facilities with higher percentages of racial/ethnic minorities, such as Black and Hispanic individuals,^8^ lower nurse staffing,^7,10^ and lower ratings for quality were associated with higher rates of SARS-CoV-2 cases and deaths.^9^ Individual resident characteristics, such as cognitive and functional status, were not evaluated in prior studies. Patients with impaired cognition or functional status may be at increased risk of SARS-CoV-2 infection because they require more assistance from staff members. There is a lack of large-scale resident-level studies among nursing homes to comprehensively describe risk factors for SARS-CoV-2 infection and outcomes.^11,12^

We used national data from long-stay nursing home residents in the US to identify risk factors for SARS-CoV-2 infections and for hospitalization and mortality after SARS-CoV-2 infections. We were interested in whether the factors associated with hospitalization and death from SARS-CoV-2 among community-dwelling populations were similar to those in long-term care.

## Methods

The cohort study was approved by the University of Texas Medical Branch institutional review board complies with the Centers for Medicare & Medicaid Services (CMS) Data Use Agreement requirements, which waived the need for informed consent for use of the study data because data were deidentified. This study followed the Strengthening the Reporting of Observational Studies in Epidemiology (STROBE) reporting guideline for cohort studies.

### Data Source

We used the Minimum Data Set (MDS) version 3.0, a federally mandated standardized resident assessment^13^ and linked Medicare claims data for 100% of US nursing home residents from January 1 to December 31, 2020, last updated on January 31, 2021. The MDS contains information on resident clinical, psychosocial, and functional characteristics. We used the Medicare Beneficiary Summary File for demographic and enrollment information, the Carrier File for physician claims, the Outpatient Standard Analytic File (SAF) for outpatient claims, the Hospital SAF for hospitalization, and skilled nursing facility claims.

### Study Population

We identified long stay residents aged 65 years and older residing in nursing homes as of April 1, 2020 (eFigure in the Supplement). Using a previously validated approach,^14,15^ we identified nursing home stays based on the MDS data and excluded any skilled nursing facility care during that stay. The Center for Disease Control and Prevention established the *International Statistical Classification of Diseases, Tenth Revision, Clinical Modification (ICD-10-CM)*^16^ code U07.1 for SARS-CoV-2 on April 1, 2020. We excluded residents if they were diagnosed with SARS-CoV-2 before April 1, 2020 using *ICD-10-CM* codes of J12.89, J20.8, J40, J22 J98.8, J80 combined with B97.29, or U07.1 to identify SARS-CoV-2. We restricted nursing home residents to those with continuous enrollment in Medicare Parts A and B with no enrollment in health maintenance organizations from April 1, 2020, until SARS-CoV-2 diagnosis, death, or the study end date on September 30, 2020. Most SARS-CoV-2 claims came from the Carrier File, followed by outpatient, inpatient, and skilled nursing facility files (eTable 1 in the Supplement). Nearly half of the patients with SARS-CoV-2 infection had 1 or 2 claims, and one-fourth had 7 or more claims (eTable 2 in the Supplement).

### Outcomes

We examined 3 outcomes: new diagnosis of SARS-CoV-2 infection until September 30, 2020, hospitalization within 30 days of SARS-CoV-2 diagnosis, and death within 30 days of SARS-CoV-2 diagnosis. For hospitalization and mortality outcomes, we followed-up patients until October 31, 2020, using claims last updated on January 31, 2021. We identified the first diagnosis of SARS-CoV-2 from the Carrier File, Outpatient SAF, Hospital SAF, or nursing facility files using *ICD-10-CM* code U07.1. Hospitalization was identified from the Hospital SAF file, and death was identified from the Medicare Beneficiary Summary File.

### Resident Characteristics

We included characteristics if there were a priori reasons why they might be associated with increased risk of SARS-CoV-2 infection, such as a condition that might necessitate more physical contact by staff or that might interfere with following instructions on social distancing. We also included characteristics associated with risk of hospitalization or death in prior studies. We identified resident characteristics from the resident’s most recent MDS assessment prior to April 1, 2020, including resident age, sex, race/ethnicity, body mass index (BMI; calculated as weight in kilograms divided by height in meters squared), cognitive function (categorized as cognitively intact and mild, moderate, and severe impairment),^17^ mood (categorized as no depression, minimal to mild depression, and moderate to severe depression),^18,19^ hallucinations or delusions or aggressive behavior (yes or no),^20^ activities of daily living score (sum of 0-4 scores for 8 activities of daily living items: 0-8 indicated no dependence; 9-16, mild dependence; 17-24, moderate dependence; and 25-32, severe dependence), use of tube or catheter (yes or no), physician prognosis of life expectancy of less than 6 months, as well as diagnosis for respiratory disease, cancer, heart disease, diabetes, renal disease, malnutrition, and neurologic conditions.

### Statistical Analysis

SARS-CoV-2 infections vary widely across geographic regions and among nursing homes.^1,2,3,9^ To describe the variation, for each outcome we constructed a 3-level logistic regression model based on resident, nursing home facility, and county level that estimated the intraclass correlation coefficient at the county and nursing home levels with and without controlling for resident characteristics.

To estimate the association of resident characteristics with SARS-CoV-2 diagnosis between April 1, 2020, and September 30, 2020, we first constructed a proportional hazards competing risk model with death as a competing risk.^21^ To account for geography and facility, we then constructed a conditional competing risk model conditioned on facility. In essence, individuals are then compared within the same facility. This controls for differences between facilities and differences between geographic areas, because each facility is nested in a geographic area.^22,23,24,25^

For hospitalization and death, the day of diagnosis was day 0, and all residents were followed-up for 30 days. We constructed a conditional competing risk model conditioned on nursing facility for hospitalization while treating death as a competing risk and conditional Cox proportional hazards regression models for death. All resident characteristics, plus the month of diagnosis of SARS-CoV-2, were included in the models. In addition, the 3-level logistic regression model provided an alternative way to control for variation due to geography and facility.

All analyses were performed using SAS Enterprise statistical software version 7.12 (SAS Institute). *P* values were 2-sided, and statistical significance was set at *P* < .05. Data were analyzed from November 22, 2020, to February 10, 2021.

## Results

This cohort study included 482 323 long-stay residents at 15 038 nursing homes. The mean (SD) age was 82.7 (9.2) years, with 326 861 (67.8%) women and 383 838 residents (79.6%) identifying as White. The SARS-CoV-2 infection rate was 28.4%. Among 137 119 residents diagnosed with COVID-19, 29 206 residents (21.3%) were hospitalized, and 26 382 residents (19.2%) died within 30 days.

### Variation in Incidence, Hospitalization, and Death Among Counties and Facilities

Table 1 shows the amount of variation in SARS-Cov-2 infection, hospitalization, and death attributed to the county and individual facilities, generated from 3-level multivariable logistic regressions models in which residents are nested in facilities and facilities nested in counties. In the null models and the models including all resident characteristics, approximately 23% of the variation in incidence was attributable to the county and 37% to the facility. County accounted for approximately 7% of the variation in risk of hospitalization and 2% in risk of death, while facility accounted for 17% of the variation in risk of hospitalization and 9% in risk of death (Table 1).

**Table 1.** Intraclass Correlation Coefficient at County and Nursing Home Facility Level From 3-Level Logistic Regression Models Estimating SARS-CoV-2 Infection, Hospitalization, and Mortality Within 30 Days of Diagnosis

Outcome	Intraclass correlation coefficient, %			
	Null model		All patient characteristics^a^	
	County (n = 2891)	Nursing home (n = 15 038)	County (n = 2891)	Nursing home (n = 15 038)
**SARS-CoV-2 infection**	**23.28**	**36.91**	**23.36**	**37.23**
**30-d**				
Acute hospitalization	5.96	16.67	7.36	16.21
Mortality	2.52	8.04	2.25	8.71

^a^ The full 3-level models for hospitalization and mortality are presented in eTable 3 in the Supplement.

### Characteristics Associated With SARS-CoV-2 Infections

Table 2 summarizes the resident characteristics associated with risk of SARS-CoV-2 diagnosis during follow up. Unadjusted SARS-Cov-2 infection rates and adjusted hazard ratios (aHRs) from a competing risk model and a conditional competing risk model that controlled for differences among nursing homes are presented.

**Table 2.** Results From a Competing Risk Regression Model and a Conditional Competing Risk Regression Model Conditioned on Nursing Home for Resident Characteristics Associated With SARS-Cov-2 Infection

Patient characteristics	Residents, No. (%)		aHR (95% CI)	
	Overall (N = 482 323)	With SARS-CoV-2 infection (n = 137 119)	Competing risk model	Conditional competing risk model (nursing home facility
Age, y				
65-70	60 709 (12.6)	19 221 (31.7)	1 [Reference]	1 [Reference]
71-75	61 066 (12.7)	18 823 (30.8)	0.99 (0.97-1.01)	1.03 (1.01-1.05)
76-80	72 091 (15.0)	21 952 (30.5)	0.99 (0.97-1.01)	1.06 (1.04-1.08)
81-85	85 897 (17.8)	24 721 (28.8)	0.93 (0.91-0.95)	1.06 (1.04-1.07)
86-90	92 252 (19.1)	25 093 (27.2)	0.88 (0.86-0.90)	1.03 (1.01-1.05)
>90	110 308 (22.9)	27 209 (24.8)	0.80 (0.79-0.82)	0.97 (0.95-0.98)
BMI				
≤18.4	31 957 (6.6)	8280 (25.9)	0.94 (0.92-0.97)	0.88 (0.86-0.90)
18.5-25	181 508 (37.6)	51 524 (28.4)	1 [Reference]	1 [Reference]
25.1-30	138 701 (28.8)	39 997 (28.8)	0.99 (0.98-1.01)	1.06 (1.05-1.07)
30.1-35	75 622 (15.7)	21 798 (28.8)	0.97 (0.96-0.99)	1.10 (1.09-1.12)
35.1-40	33 248 (6.9)	9502 (28.6)	0.95 (0.93-0.97)	1.13 (1.10-1.15)
40.1-45	13 954 (2.9)	3980 (28.5)	0.94 (0.91-0.97)	1.15 (1.12-1.19)
>45	7333 (1.5)	2038 (27.8)	0.90 (0.86-0.95)	1.19 (1.15-1.24)
Sex				
Women	326 861 (67.8)	90 501 (27.7)	1 [Reference]	1 [Reference]
Men	155 462 (32.2)	46 618 (30.0)	1.03 (1.01-1.04)	1.08 (1.06-1.09)
Race/ethnicity				
White	383 838 (79.6)	98 532 (25.7)	1 [Reference]	1 [Reference]
Black	60 810 (12.6)	23 698 (39.0)	1.56 (1.54-1.58)	1.04 (1.03-1.06)
Asian	9819 (2.0)	3899 (39.7)	1.63 (1.58-1.69)	1.07 (1.03-1.11)
Hispanic or Latino	24 732 (5.1)	10 346 (41.8)	1.72 (1.69-1.76)	1.07 (1.05-1.10)
Other	3124 (0.7)	644 (20.6)	0.74 (0.68-0.79)	1.02 (0.94-1.11)
Cognitive function				
Cognitively intact	141 160 (29.3)	39 819 (28.2)	1 [Reference]	1 [Reference]
Impaired				
Mildly	117 292 (24.3)	33 798 (28.8)	1.02 (1.01-1.04)	1.02 (1.01-1.03)
Moderately	170 815 (35.4)	49 373 (28.9)	1.03 (1.02-1.05)	1.02 (1.01-1.03)
Severely	53 056 (11.0)	14 129 (26.6)	0.94 (0.92-0.96)	0.98 (0.96-0.99)
Mood, depression				
None	239 232 (49.6)	74 403 (31.1)	1 [Reference]	1 [Reference]
Minimal or mild	210 691 (43.7)	53 947 (25.6)	0.85 (0.84-0.86)	0.99 (0.98-1.01)
Moderate or severe	32 400 (6.7)	8769 (27.1)	0.93 (0.91-0.95)	0.95 (0.93-0.98)
Hallucinations or aggressive behavior				
No	393 328 (81.6)	114 163 (29.0)	1 [Reference]	1 [Reference]
Yes	88 995 (18.5)	22 956 (25.8)	0.90 (0.89-0.91)	1.00 (0.98-1.02)
Functional impairment^a^				
None	42 235 (8.8)	10 603 (25.1)	1 [Reference]	1 [Reference]
Mild	83 533 (17.3)	24 778 (29.7)	1.23 (1.21-1.26)	1.05 (1.03-1.07)
Moderate	222 813 (46.2)	63 655 (28.6)	1.24 (1.22-1.27)	1.03 (1.01-1.05)
Severe	133 742 (27.7)	38 083 (28.5)	1.22 (1.19-1.25)	0.94 (0.92-0.96)
Use of catheter or tube^b^				
No	433 164 (89.8)	122 961 (28.4)	1 [Reference]	1 [Reference]
Yes	49 159 (10.2)	14 158 (28.8)	0.96 (0.95-0.98)	0.96 (0.94-0.98)
Prognosis of <6 mos				
No	451 116 (93.5)	132 115 (29.3)	1 [Reference]	1 [Reference]
Yes	31 207 (6.5)	5004 (16.0)	0.54 (0.52-0.55)	0.55 (0.53-0.56)
Cancer				
No	445 896 (92.5)	127 549 (28.6)	1 [Reference]	1 [Reference]
Yes	36 427 (7.6)	9570 (26.3)	0.95 (0.93-0.97)	0.96 (0.94-0.98)
Heart disease^c^				
No	74 328 (15.4)	19 798 (26.6)	1 [Reference]	1 [Reference]
Yes	407 995 (84.6)	117 321 (28.8)	1.06 (1.05-1.08)	1.01 (0.99-1.02)
Renal disease^d^				
None	391 673 (81.2)	111 339 (28.4)	1 [Reference]	1 [Reference]
Any	90 650 (18.8)	25 780 (28.4)	0.98 (0.97-0.99)	1.03 (1.02-1.05)
Diabetes				
No	319 219 (66.2)	87 573 (27.4)	1 [Reference]	1 [Reference]
Yes	163 104 (33.8)	49 546 (30.4)	1.03 (1.01-1.04)	1.02 (1.01-1.03)
Neurologic conditions^d^				
No	380 162 (78.8)	106 476 (28.0)	1 [Reference]	1 [Reference]
Yes	102 161 (21.2)	30 643 (30.0)	0.97 (0.95-0.98)	1.00 (0.99-1.02)
Malnutrition				
No	441 764 (91.6)	124 988 (28.3)	1 [Reference]	1 [Reference]
Yes	40 559 (8.4)	12 131 (29.9)	1.05 (1.03-1.07)	0.97 (0.95-0.99)
Respiratory conditions^e^				
No	337 538 (70.0)	97 589 (28.9)	1 [Reference]	1 [Reference]
Yes	144 785 (30.0)	39 530 (27.3)	0.96 (0.95-0.97)	1.01 (0.99-1.02)

Abbreviations: aHR, adjusted hazards ratio; BMI, body mass index (calculated as weight in kilograms divided by height in meters squared).

^a^ Functional impairment was categorized as no dependence (activities of daily living score, 0-8), mild (activities of daily living score, 9-16), moderate (activities of daily living score, 17-24), and severe dependence (activities of daily living score, 25-32).

^b^ Use of catheter or tube included indwelling catheter, parenteral intravenous line, and feeding tube.

^c^ Heart disease included coronary artery disease, heart failure, and hypertension.

^d^ Neurologic conditions included stroke, hemiplegia, and paraplegia.

^e^ Respiratory conditions included chronic obstructive pulmonary disease, respiratory failure, and shortness of breath.

The magnitude of the HR for SARS-CoV-2 infection changed substantially for some variables in the conditional competing risk models that controlled for differences among nursing homes, compared with the competing risk model. For example, Black residents (aHR, 1.56; 95% CI, 1.54-1.58), Hispanic residents (aHR, 1.72; 95% CI, 1.59-1.76) and Asian residents (aHR, 1.63; 95% CI, 1.58-1.69) had higher risk of SARS-CoV-2 infections in the competing risk model that did not control for the variation in risk among facilities or counties. In the model conditioned on facility, which controlled for differences among facilities and also (indirectly) among counties, the aHR was reduced to 1.04 (95% CI, 1.03-1.06) for Black residents, 1.07 (95% CI, 1.05-1.10) for Hispanic residents, and 1.07 (1.03-1.11) for Asian residents.

In the conditional model, there was a monotonic association between BMI and risk of infection (eg, compared with BMI 18.1-25, BMI<18.5: aHR, 0.88; 95% CI, 0.86-0.90 vs BMI>45: aHR, 1.19; 95% CI, 1.15-1.24). Characteristics that might indicate need for more care and staff contact, such as severe cognitive or functional impairment or having a feeding tube, intravenous line, or catheter, were associated with increased risk of infection. An estimated poor life expectancy (aHR, 0.55; 95% CI, 0.53-0.56) was the only characteristic other than BMI and race/ethnicity that was associated with a greater than 6% difference in risk of SARS-CoV-2 diagnosis.

### Characteristics Associated With Hospitalization After a Diagnosis of SARS-CoV-2

As shown in Table 3, the unadjusted rates and the adjusted risk of hospitalization from a conditional competing risk model increased with increasing BMI (eg, BMI>45 vs BMI 18.5-25: aHR, 1.40; 95% CI, 1.28-1.52). Men (aHR, 1.32; 95% CI, 1.29-1.35), Black residents (aHR, 1.28; 95% CI, 1.24-1.32), Hispanic residents (aHR, 1.20; 95% CI, 1.15-1.26), and Asian residents (aHR, 1.46; 95% CI, 1.36-1.57) diagnosed with SARS-CoV-2 had higher risks of hospitalization. Hospitalization risk was higher with increasing cognitive impairment or functional impairment. Several comorbidities, including renal disease (aHR, 1.21; 95% CI, 1.18-1.24), diabetes (aHR, 1.16; 95% CI, 1.13-1.18), and respiratory conditions (aHR, 1.14; 95% CI, 1.11-1.16), were associated with increased risk of hospitalization. The adjusted hazard of hospitalization after SARS-CoV-2 diagnosis declined from April through September (eg, compared with April, May: aHR, 0.50; 95% CI, 0.48-0.52; September: aHR, 0.21; 95% CI, 0.20-0.23).

**Table 3.** Results from a Conditional Competing Risk Model Conditioned on Facility for Resident Characteristics Associated With Acute Hospitalization 30 Days After SARS-CoV-2 Infection

Characteristic	Residents, No. (%)		aHR (95% CI)
	With SARS-CoV-2 infection (n = 137 119)	Hospitalized for SARS-CoV-2^a^ (n = 29 204)	
Age, y			
65-70	19 221 (14.0)	4492 (23.4)	1 [Reference]
71-75	18 823 (13.7)	4617 (24.5)	1.07 (1.03-1.11)
76-80	21 952 (16.0)	5144 (23.4)	1.08 (1.04-1.12)
81-85	24 721 (18.0)	5585 (22.6)	1.14 (1.10-1.19)
86-90	25 093 (18.3)	5002 (19.9)	1.08 (1.04-1.13)
>90	27 309 (19.9)	4364 (16.0)	0.95 (0.91-0.99)
BMI			
≤18.4	8280 (6.0)	1446 (17.5)	0.93 (0.89-0.98)
18.5-25	51 524 (37.6)	10 387 (20.2)	1 [Reference]
25.1-30	39 997 (29.2)	8656 (21.6)	1.04 (1.02-1.07)
30.1-35	21 798 (15.9)	4941 (22.7)	1.12 (1.08-1.16)
35.1-40	9502 (6.9)	2227 (23.4)	1.16 (1.11-1.21)
40.1-45	3980 (2.9)	1008 (25.3)	1.24 (1.16-1.32)
>45	2038 (1.5)	239 (26.5)	1.40 (1.28-1.52)
Sex			
Women	90 501 (66.0)	16 797 (18.6)	1 [Reference]
Men	46 618 (34.0)	12 407 (26.6)	1.32 (1.29-1.35)
Race/ethnicity			
White	98 532 (71.9)	17 727 (18.0)	1 [Reference]
Black	23 698 (17.3)	7293 (30.8)	1.28 (1.24-1.32)
Asian	3899 (2.8)	1067 (27.4)	1.46 (1.36-1.57)
Hispanic or Latino	10 346 (7.6)	2969 (28.7)	1.20 (1.15-1.26)
Other	644 (0.5)	148 (23.0)	1.09 (0.92-1.31)
Cognitive function			
Cognitively intact	39 819 (29.0)	8607 (21.6)	1 [Reference]
Impaired			
Mildly	33 798 (24.7)	7304 (21.6)	1.01 (0.98-1.04)
Moderately	49 373 (36.0)	10 399 (21.1)	1.06 (1.03-1.09)
Severely	14 129 (10.3)	2894 (20.5)	1.06 (1.01-1.10)
Mood, depression			
None	74 403 (54.3)	15 925 (20.6)	1 [Reference]
Minimal or mild	53 947 (39.3)	11 136 (21.4)	1.07 (1.05-1.10)
Moderate or severe	8769 (6.4)	2143 (24.4)	1.06 (1.01-1.12)
Hallucinations or aggressive behavior			
No	114 163 (83.3)	24 494 (21.5)	1 [Reference]
Yes	22 956 (16.7)	4710 (20.5)	1.02 (0.98-1.05)
Functional impairment^b^			
None	10 603 (7.7)	2044 (19.3)	1 [Reference]
Mild	24 778 (18.1)	5086 (20.5)	1.08 (1.03-1.14)
Moderate	63 655 (46.4)	13 490 (21.2)	1.15 (1.10-1.21)
Severe	38 083 (27.8)	8584 (22.5)	1.15 (1.10-1.22)
Use of catheter or tube^c^			
No	122 961 (89.7)	25 359 (20.6)	1 [Reference]
Yes	14 158 (10.3)	3845 (27.2)	1.21 (1.16-1.25)
Prognosis of <6 mos			
No	132 115 (96.4)	28 848 (21.9)	1 [Reference]
Yes	5004 (3.7)	356 (7.1)	0.34 (0.31-0.37)
Cancer			
No	127 549 (93.0)	27 013 (21.2)	1 [Reference]
Yes	9570 (7.0)	2191 (22.9)	1.05 (1.01-1.09)
Heart disease^d^			
No	19 798 (14.4)	3356 (17.0)	1 [Reference]
Yes	117 321 (85.6)	25 848 (22.0)	1.12 (1.08-1.16)
Renal disease			
None	111 339 (81.2)	22 397 (20.1)	1 [Reference]
Any	25 780 (18.8)	6807 (26.4)	1.21 (1.18-1.24)
Diabetes			
No	87 573 (63.9)	16 425 (18.8)	1 [Reference]
Yes	49 546 (36.1)	12 779 (25.8)	1.16 (1.13-1.18)
Neurologic conditions^e^			
No	106 476 (77.7)	21 897 (20.6)	1 [Reference]
Yes	30 643 (22.4)	7307 (23.9)	1.01 (0.98-1.03)
Malnutrition			
No	124 988 (91.2)	26 444 (21.2)	1 [Reference]
Yes	12 131 (8.9)	2760 (22.8)	1.03 (0.99-1.08)
Respiratory conditions^f^			
No	97 589 (71.2)	19 694 (20.2)	1 [Reference]
Yes	39 530 (28.8)	9510 (24.1)	1.14 (1.11-1.16)
Month of SARS-CoV-2 infection			
April	35 132 (25.6)	11 041 (31.4)	1 [Reference]
May	27 775 (20.3)	5549 (20.0)	0.50 (0.48-0.52)
June	20 197 (14.7)	2603 (12.9)	0.28 (0.27-0.30)
July	23 298 (17.0)	4430 (19.0)	0.29 (0.27-0.31)
August	16 342 (11.9)	3388 (20.7)	0.28 (0.26-0.30)
September	14 375 (10.5)	2193 (15.3)	0.21 (0.20-0.23)

Abbreviations: aHR, adjusted hazards ratio; BMI, body mass index (calculated as weight in kilograms divided by height in meters squared).

^a^ Values represent row percentage. These are unadjusted rates and did not account for dropout or censoring.

^b^ Functional impairment was categorized as no dependence (activities of daily living score, 0-8), mild (activities of daily living score, 9-16), moderate (activities of daily living score, 17-24), and severe dependence (activities of daily living score, 25-32).

^c^ Use of catheter or tube included indwelling catheter, parenteral intravenous line, and feeding tube.

^d^ Heart disease included coronary artery disease, heart failure, and hypertension.

^e^ Neurologic conditions included stroke, hemiplegia, and paraplegia.

^f^ Respiratory conditions included chronic obstructive pulmonary disease, respiratory failure, and shortness of breath.

Results from the 3-level logistic regression models assessing resident characteristics associated with risk of hospitalization were in accordance with the main analysis (eTable 3 in the Supplement).

### Characteristics Associated With Mortality After SARS-CoV-2

As shown in Table 4, risk of death in the 30 days after SARS-CoV-2 infection increased with age (age >90 years vs 65-70 years: aHR, 2.55; 95% CI, 2.44-2.67). There was no association of mortality with high BMI (BMI>45: aHR, 1.05; 95% CI, 0.95-1.16), while mortality was increased with BMI less than 18.5 (aHR, 1.19; 95% CI, 1.14-1.24) compared with BMI of 18.5 to 25. Men were at higher risk of death (aHR, 1.57; 95% CI, 1.53-1.61). Black (aHR, 0.99; 95% CI, 0.95-1.02) and Hispanic (aHR, 0.97; 95% CI, 0.93-1.02) race/ethnicity were not associated with mortality after a SARS-CoV-2 diagnosis, while Asian race/ethnicity (aHR, 1.19; 95% CI, 1.10-1.28) had higher risk. Risk of mortality increased with increasing cognitive dysfunction (eg, compared with no impairment, moderately impaired: aHR, 1.45; 95% CI, 1.41-1.50; severely impaired: aHR, 1.79; 95% CI, 1.71-1.86) and impaired functional status (eg, compared with functional independence, moderately dependent: aHR, 1.55; 95% CI, 1.47-1.64; fully dependent: aHR, 1.94; 95% CI, 1.83-2.05). Similar to the findings with risk of hospitalization, risk of mortality after a diagnosis of SARS-CoV-2 declined by half from April to May, then continued to decline through September. Results from 3-level logistic regression models showed similar findings to the main analysis for the association of patient characteristics with mortality after a SARS-CoV-2 diagnosis (eTable 3 in the Supplement).

**Table 4.** Results from a Conditional Competing Risk Model Conditioned on Facility for Resident Characteristics Associated With Mortality 30 Days After SARS-CoV-2 Infection

Characteristic	Residents, No. (%)		aHR (95% CI)
	With SARS-CoV-2 infection (n = 137 119)	SARS-CoV-2–related mortality (n = 26 384)^a^	
Age, y			
65-70	19 221 (14.0)	2274 (11.8)	1 [Reference]
71-75	18 823 (13.7)	2923 (15.5)	1.30 (1.24-1.36)
76-80	21 952 (16.0)	3907 (17.8)	1.51 (1.44-1.58)
81-85	24 721 (18.0)	4931 (20.0)	1.76 (1.69-1.84)
86-90	25 093 (18.3)	5494 (21.9)	2.06 (1.96-2.15)
>90	27 309 (19.9)	6855 (25.1)	2.55 (2.44-2.67)
BMI			
≤18.4	8280 (6.0)	1941 (23.4)	1.19 (1.14-1.24)
18.5-25	51 524 (37.6)	10 774 (20.9)	1 [Reference]
25.1-30	39 997 (29.2)	7467 (18.7)	0.90 (0.87-0.92)
30.1-35	21 798 (15.9)	3770 (17.3)	0.90 (0.87-0.93)
35.1-40	9502 (6.9)	1527 (16.1)	0.90 (0.86-0.95)
40.1-45	3980 (2.9)	584 (14.7)	0.89 (0.83-0.96)
>45	2038 (1.5)	321 (15.8)	1.05 (0.95-1.16)
Sex			
Women	90 501 (66.0)	15 574 (17.2)	1 [Reference]
Men	46 618 (34.0)	10 810 (23.2)	1.57 (1.53-1.61)
Race/ethnicity			
White	98 532 (71.9)	18 548 (18.8)	1 [Reference]
Black	23 698 (17.3)	4690 (19.8)	0.99 (0.95-1.02)
Asian	3899 (2.8)	853 (21.9)	1.19 (1.10-1.28)
Hispanic or Latino	10 346 (7.6)	2133 (20.6)	0.97 (0.93-1.02)
Other	644 (0.5)	160 (24.8)	1.35 (1.15-1.59)
Cognitive function			
Cognitively intact	39 819 (29.0)	5481 (13.8)	1 [Reference]
Impaired			
Mildly	33 798 (24.7)	5924 (17.5)	1.17 (1.14-1.21)
Moderately	49 373 (36.0)	11 099 (22.5)	1.45 (1.41-1.50)
Severely	14 129 (10.3)	3880 (27.5)	1.79 (1.71-1.86)
Mood, depression			
None	74 403 (54.3)	13 750 (18.5)	1 [Reference]
Minimal or mild	53 947 (39.3)	10 762 (20.0)	1.08 (1.05-1.11)
Moderate or severe	8769 (6.4)	1872 (21.4)	1.15 (1.09-1.22)
Hallucinations or aggressive behavior			
No	114 163 (83.3)	21 452 (18.8)	1 [Reference]
Yes	22 956 (16.7)	4932 (21.5)	1.12 (1.09-1.16)
Functional impairment^b^			
None	10 603 (7.7)	1235 (11.7)	1 [Reference]
Mild	24 778 (18.1)	3698 (14.9)	1.24 (1.17-1.32)
Moderate	63 655 (46.4)	12 335 (19.4)	1.55 (1.47-1.64)
Severe	38 083 (27.8)	9116 (24.0)	1.94 (1.83-2.05)
Use of catheter or tube^a^			
No	122 961 (89.7)	23 367 (19.0)	1 [Reference]
Yes	14 158 (10.3)	3017 (21.3)	1.03 (0.99-1.07)
Prognosis of <6 mos			
No	132 115 (96.4)	24 980 (18.9)	1 [Reference]
Yes	5004 (3.7)	1404 (28.1)	1.47 (1.40-1.55)
Cancer			
No	127 549 (93.0)	24 277 (19.0)	1 [Reference]
Yes	9570 (7.0)	2107 (22.0)	1.17 (1.12-1.21)
Heart disease^c^			
No	19 798 (14.4)	3519 (17.8)	1 [Reference]
Yes	117 321 (85.6)	22 865 (19.5)	1.07 (1.04-1.11)
Renal disease			
None	111 339 (81.2)	20 682 (18.6)	1 [Reference]
Any	25 780 (18.8)	5702 (22.1)	1.23 (1.20-1.26)
Diabetes			
No	87 573 (63.9)	16 519 (18.9)	1 [Reference]
Yes	49 546 (36.1)	9865 (19.9)	1.16 (1.13-1.19)
Neurologic conditions^d^			
No	106 476 (77.7)	20 700 (19.4)	1 [Reference]
Yes	30 643 (22.4)	5684 (18.6)	0.94 (0.92-0.97)
Malnutrition			
No	124 988 (91.2)	24 011 (19.2)	1 [Reference]
Yes	12 131 (8.9)	2373 (19.6)	1.01 (0.97-1.06)
Respiratory conditions^e^			
No	97 589 (71.2)	18 686 (19.2)	1 [Reference]
Yes	39 530 (28.8)	7698 (19.5)	1.11 (1.08-1.14)
Month of SARS-CoV-2 infection			
April	35 132 (25.6)	10 498 (29.9)	1 [Reference]
May	27 775 (20.3)	4839 (17.4)	0.52 (0.50-0.54)
June	20 197 (14.7)	2165 (10.7)	0.35 (0.33-0.37)
July	23 298 (17.0)	3702 (15.9)	0.37 (0.35-0.39)
August	16 342 (11.9)	2905 (17.8)	0.36 (0.33-0.38)
September	14 375 (10.5)	2275 (15.8)	0.29 (0.27-0.32)

Abbreviations: aHR, adjusted hazards ratio; BMI, body mass index (calculated as weight in kilograms divided by height in meters squared).

^a^ Functional impairment was categorized as no dependence (activities of daily living score, 0-8), mild (activities of daily living score, 9-16), moderate (activities of daily living score, 17-24), and severe dependence (activities of daily living score, 25-32).

^b^ Values represent row percentage. These are unadjusted rates and did not account for dropout or censoring.

^c^ Use of catheter/tube included indwelling catheter, parenteral intravenous line, and feeding tube.

^d^ Heart disease included coronary artery disease, heart failure, and hypertension.

^e^ Neurologic conditions included stroke, hemiplegia, and paraplegia.

## Discussion

In this cohort study of more than 480 000 US long-stay nursing home residents, we found that the risk of infection was primarily associated with geography and the particular nursing home facility, with minimal contribution of individual characteristics of residents. To our knowledge, this is the first national study of long-stay nursing home residents. Previous studies have reported that community factors, such as percentage of foreign-born, household size, and job type, were associated with increased risk of SARS-CoV-2 infection.^26^ Also, nursing home characteristics, such as size, percentage of Black, Hispanic, or Asian population, percentage of residents enrolled in Medicaid, and lower staffing, were associated with increased SARS-CoV-2 infections.^7,8,9,10,27,28^ Risk of hospitalization and risk of death in the 30 days after a SARS-CoV-2 diagnosis were highest in April, with steep declines thereafter, consistent with prior reports.^29,30^

Resident characteristics played a large role in risk of hospitalization and death. With individual characteristics, such as age, race/ethnicity, cognitive status, and functional status, there was a large divergence between the magnitude of their association with hospitalization vs the magnitude of association with mortality. With race/ethnicity and BMI, the magnitude of risk of hospitalization were considerable higher than magnitude of risk for mortality. With other characteristics, the magnitude of risk for mortality was substantially higher than the magnitude of risk for hospitalization.

The interpretation of the divergence between risks of hospitalization vs risks of mortality is complex. In studies of community populations, the risks of hospitalization and the risks of death associated with increasing age tend to parallel each other, consistent with the concept that decisions for hospitalization are influenced by clinical judgement on risk of death.^5,31,32,33^ A similar pattern is seen with other diseases, such as influenza.^34^ In contrast, there was an inconsistent association of risk of hospitalization with increasing age among US nursing home residents diagnosed with SARS-CoV-2. This may represent resident or family preference to avoid hospitalization, or triaging decisions in areas where hospital beds were scarce, or lack of appropriate clinical evaluation and prognostication in facilities overwhelmed by the pandemic, or perhaps some combination of these or other explanations. Understanding this phenomenon may require a qualitative approach.

In contrast, residents who were Black, Hispanic, or Asian had substantially higher risk of hospitalization after SARS-CoV-2 diagnosis, but the risk for mortality was very close to that of White residents. For example, after controlling for differences among nursing homes, Black residents were at 28% higher risk of hospitalization but not at increased risk of death. Studies of community residents have also found higher hospitalization rates for Black individuals,^5,32,33^ with little or no differences in mortality.^5,33^ Consistent with prior studies, we found that Asian residents were at increased risk of hospitalization and mortality.^35,36^ A higher prevalence of heart disease, as well as language barriers, may contribute to disparity among Asian individuals.^36,37^

We should emphasize that all of these results, including ours, are from analyses that controlled for other resident characteristics and that also controlled for the individual nursing home facility using conditional models. Other investigators have reported an overall increase in SARS-CoV-2 mortality among Black nursing home residents that is explained partly by geographic location (ie, areas of the US with high initial infection rates were often also areas with higher Black populations) and partly by nursing home quality (eg, residents in facilities with a high proportion of Black residents have worse outcomes from SARS-CoV-2 and other conditions, independent of resident race).^8,9^

There was a similar divergence between hospitalization risk and risk of mortality with increasing BMI. An association of BMI with death after SARS-CoV-2 infections was found in community populations, while we found no association of mortality risk with increasing BMI in nursing home residents.^38,39,40,41^ With Black, Hispanic, or Asian race/ethnicity and elevated BMI, there were early reports suggesting elevated mortality risks. This may have contributed to an increased clinical sensitivity to those factors. Indeed, the increase in hospitalization rates in Black, Hispanic, or Asian nursing home residents and in residents who were obese may have contributed to the lower mortality rates.

We found an association of increasing cognitive impairment with death after SARS-CoV-2 diagnosis, similar to studies in other settings.^4,42,43^ However, there were inconsistent associations of cognitive function with hospitalization. Residents with cognitive impairment may be unable to make decisions for their treatment and may lack family member support owing to nursing facility and hospital restrictions.^42,43^ These residents also may be more likely to have advance directives precluding invasive measures. The prevalence of comorbidities was much greater in nursing home residents compared with the general population, but the associations of comorbidities with SARS-CoV-2 infection, hospitalization, and mortality were similar to community studies.^44,45^

### Limitations

The study has several limitations. First, we evaluated rate of clinical diagnosis of SARS-CoV-2 infections, not the rate of actual infections. The intensity of diagnostic evaluation for SARS-CoV-2 may differ by patient characteristics, leading to underrecognition of disease. This may be the reason why residents with an estimated life expectancy of less than 6 months had a lower risk of diagnosis with SARS-CoV-2. Conversely, the *ICD-10-CM* code for SARS-CoV-2 may have been used in residents without the disease. Second, the study findings cannot be generalized to residents without Medicare or with Medicare Advantage. In 2020, 36% of Medicare beneficiaries were enrolled in a Medicare Advantage plan, and the share of beneficiaries in Medicare Advantage plans ranges widely across states.^46^ Third, we did not explore what nursing home characteristics were associated with outcomes. Fourth, there was no official *ICD-10-CM* code for SARS-CoV-2 prior to April 1, 2020, and that code may not have been universally used, particularly in April. Fifth, the SARS-CoV-2 pandemic may have disrupted data submission, and there may be delays in submission of Medicare claims.^47^ However, we analyzed Medicare data updated as of January 31, 2021, while the hospitalization and death outcomes were through October 31, 2020. Sixth, we had no data on completion and content of advance directives, which presumably influenced the extent of treatment received.

## Conclusions

In this national cohort study of long-stay nursing home residents, risk of SARS-CoV-2 was associated with the geographic area and particularly by facility, while risk of hospitalization and death after SARS-CoV-2 was associated with individual resident characteristics. We identified novel risk factors, such as impaired cognition and physical functioning. For many resident characteristics, there are substantial differences in risk of hospitalization vs mortality. This may represent resident preferences, triaging decisions, or inadequate assessment of risk of death.

## Notes

Supplement. **eFigure.** Cohort Selection Flowchart **eTable 1.** SARS-CoV-2 Diagnoses From Different Medicare Claims **eTable 2.** Number of SARS-CoV-2 Claims Per Resident **eTable 3.** Resident Characteristics Associated With Acute Hospitalization and Mortality 30 Days After SARS-CoV-2 Infection
