# Supplementary material for: Risk Factors Associated With SARS-CoV-2 Infections, Hospitalization, and Mortality Among US Nursing Home Residents
Source: JAMA Netw Open. 2021 Mar 31;4(3):e216315. doi: 10.1001/jamanetworkopen.2021.6315 (PMC8013796; doi:10.1001/jamanetworkopen.2021.6315)
Supplement: Supplement. — eFigure. Cohort Selection Flowchart eTable 1. SARS-CoV-2 Diagnoses From Different Medicare Claims eTable 2. Number of SARS-CoV-2 Claims Per Resident eTable 3. Resident Characteristics Associated With Acute Hospitalization and Mortality 30 Days After SARS-CoV-2 Infection [file jamanetwopen-e216315-s001.pdf]

Supplemental Online Content

Mehta HB, Li S, Goodwin JS. Risk factors associated with SARS-CoV-2 infections, hospitalization, and mortality among US nursing home residents. *JAMA Netw Open*. 2021;4(3):e216315. doi:10.1001/jamanetworkopen.2021.6315

eFigure. Cohort Selection Flowchart
eTable 1. SARS-CoV-2 Diagnoses From Different Medicare Claims
eTable 2. Number of SARS-CoV-2 Claims Per Resident
eTable 3. Resident Characteristics Associated With Acute Hospitalization and Mortality 30 Days After SARS-CoV-2 Infection

This supplemental material has been provided by the authors to give readers additional information about their work.

© 2021 Mehta HB et al. *JAMA Network Open.*

eFigure. Cohort Selection Flowchart

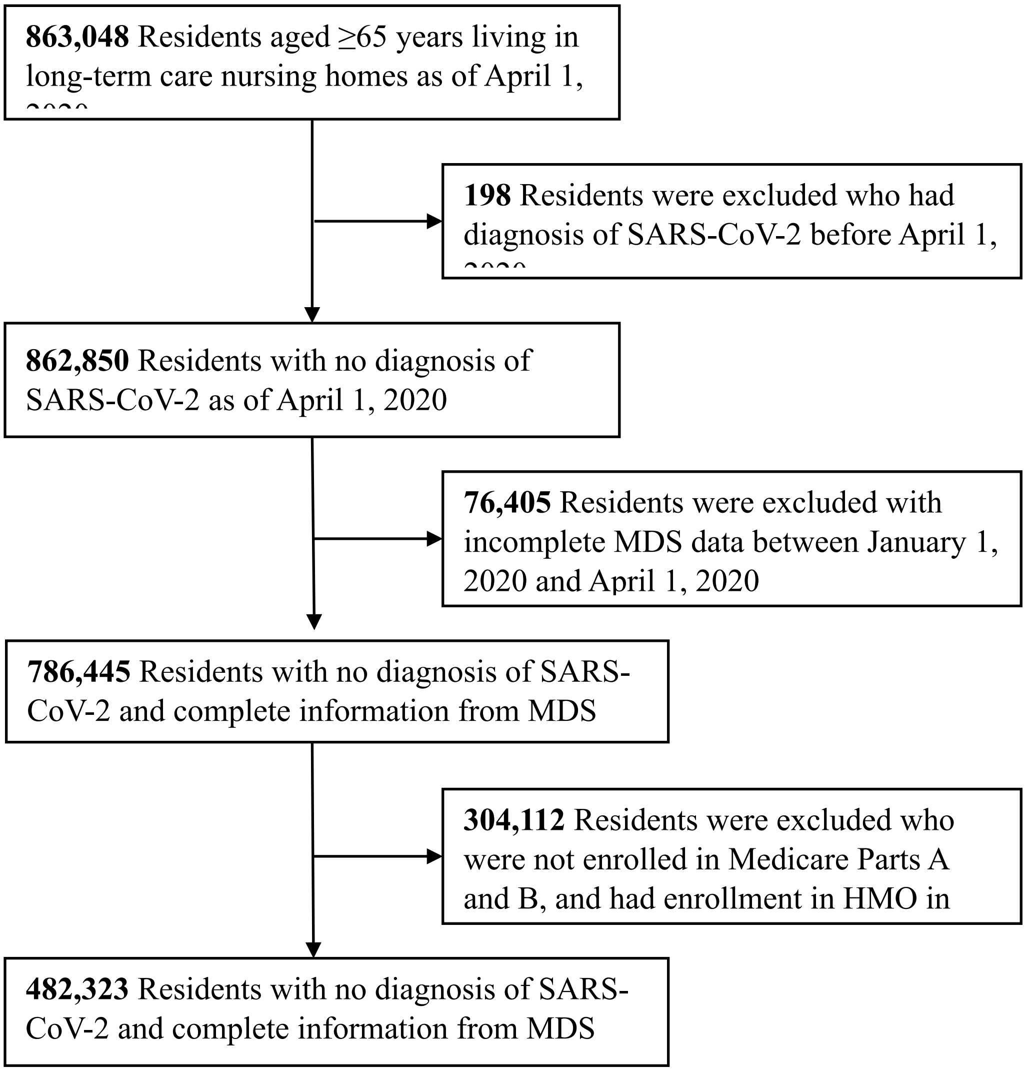

Abbreviations: SARS-CoV-2, severe acute respiratory syndrome coronavirus 2; MDS, minimum data set; HMO, health maintenance organization

© 2021 Mehta HB et al. *JAMA Network Open.*

eTable 1. SARS-CoV-2 Diagnoses From Different Medicare Claims

	Carrier claim	Outpatient claim	Inpatient claim	SNF claims
SARS-CoV-2 diagnosis in claims	8,127,623 (81.91%)	843,253 (8.50%)	493,663 (4.97%)	458,415 (4.62%)
Earliest SARS-CoV-2 claim for each patient	1,285,238 (77.21%)	281,557 (16.91%)	35,802 (2.15%)	62,025 (3.73%)

© 2021 Mehta HB et al. *JAMA Network Open.*

eTable 2. Number of SARS-CoV-2 Claims Per Resident

Number of claims	Number of patients (%)
1	601,679 (36.15%)
2	261,609 (15.72%)
3	136,916 (8.23%)
4	96,038 (5.77%)
5	73,114 (4.39%)
6	60,518 (3.64%)
7	51,448 (3.09%)
8 and above	383,290 (23.03%)

© 2021 Mehta HB et al. *JAMA Network Open.*

eTable 3. Resident Characteristics Associated With Acute Hospitalization and Mortality 30 Days After SARS-CoV-2 Infection

Results from three-level logistic regression models (resident, nursing home, county).[a]

	**30-day hospitalization, adjusted hazard ratio (95% CI)**	**30-day mortality, adjusted hazard ratio (95% CI)**
Overall		
**Age, years**		
65-70	Ref	Ref
71-75	1.13 (1.07, 1.19)	1.39 (1.31, 1.48)
76-80	1.13 (1.07, 1.19)	1.65 (1.55, 1.75)
81-85	1.17 (1.11, 1.23)	1.96 (1.85, 2.08)
86-90	1.07 (1.02, 1.13)	2.32 (2.18, 2.46)
>90	0.91 (0.86, 0.96)	2.94 (2.77, 3.13)
**Body mass index, kg/m^2**		
<=18.4	0.89 (0.84, 0.96)	1.17 (1.11, 1.25)
18.5-25	Ref	Ref
25.1-30	1.06 (1.02, 1.10)	0.92 (0.88, 0.95)
30.1-35	1.15 (1.10, 1.20)	0.94 (0.90, 0.99)
35.1-40	1.18 (1.11, 1.25)	0.97 (0.91, 1.03)
40.1-45	1.32 (1.21, 1.43)	0.97 (0.88, 1.06)
>45	1.50 (1.34, 1.68)	1.22 (1.07, 1.39)
**Sex**		
Female	Ref	Ref
Male	1.44 (1.39, 1.48)	1.82 (1.76, 1.88)
**Race/ethnicity**		
White	Ref	Ref
Black	1.49 (1.43, 1.55)	1.02 (0.98, 1.07)

© 2021 Mehta HB et al. *JAMA Network Open.*

Asian	1.70 (1.54, 1.87)	1.25 (1.13, 1.37)
Hispanic or Latino	1.34 (1.26, 1.43)	1.04 (0.98, 1.11)
Others	1.32 (1.06, 1.64)	1.54 (1.26, 1.89)
**Cognitive function**		
Cognitively intact	Ref	Ref
Mildly impaired	1.01 (0.97, 1.05)	1.20 (1.15, 1.25)
Moderately impaired	1.08 (1.04, 1.12)	1.55 (1.49, 1.61)
Severely impaired	1.05 (0.99, 1.12)	1.99 (1.88, 2.11)
**Mood**		
No depression	Ref	Ref
Minimal or Mild depression	1.07 (1.03, 1.11)	1.07 (1.04, 1.11)
Moderate or severe Depression	1.08 (1.01, 1.16)	1.10 (1.02, 1.17)
**Hallucinations/ aggressive behavior**		
No	Ref	Ref
Yes	1.02 (0.98, 1.06)	1.15 (1.11, 1.20)
**Functional impairment**[b]		
None	Ref	Ref
Mild	1.09 (1.02, 1.16)	1.21 (1.12, 1.30)
Moderate	1.15 (1.08, 1.22)	1.52 (1.42, 1.62)
Severe	1.16 (1.09, 1.24)	1.91 (1.77, 2.05)
**Use of catheter/tube**[c]		
No	Ref	Ref
Yes	1.25 (1.19, 1.31)	0.98 (0.94, 1.03)
**Prognosis of less than 6 months**		
No	Ref	Ref
Yes	0.28 (0.25, 0.32)	1.43 (1.34, 1.54)
**Cancer**		

© 2021 Mehta HB et al. *JAMA Network Open.*

No	Ref	Ref
Yes	1.05 (0.99, 1.11)	1.13 (1.07, 1.19)
**Heart Disease[d]**		
No	Ref	Ref
Yes	1.14 (1.09, 1.20)	1.08 (1.03, 1.13)
**Renal disease**		
None	Ref	Ref
Any	1.28 (1.24, 1.33)	1.23 (1.18, 1.27)
**Diabetes**		
No	Ref	Ref
Yes	1.22 (1.18, 1.25)	1.18 (1.14, 1.22)
**Neurologic conditions[e]**		
No	Ref	Ref
Yes	1.00 (0.96, 1.04)	0.94 (0.90, 0.97)
**Malnutrition**		
No	Ref	Ref
Yes	1.04 (0.98, 1.10)	0.98 (0.92, 1.03)
**Respiratory conditions[f]**		
No	Ref	Ref
Yes	1.20 (1.16, 1.24)	1.11 (1.08, 1.15)
**Month of SARS-CoV-2 infection**		
April	Ref	Ref
May	0.43 (0.41, 0.45)	0.43 (0.41, 0.45)
June	0.23 (0.21, 0.24)	0.25 (0.24, 0.27)
July	0.31 (0.29, 0.33)	0.38 (0.35, 0.40)
August	0.33 (0.31, 0.36)	0.41 (0.38, 0.43)
September	0.25 (0.23, 0.27)	0.35 (0.33, 0.38)

© 2021 Mehta HB et al. *JAMA Network Open.*

Abbreviations: ADL, activities of daily living; aHR, adjusted hazards ratio; CI, confidence interval; SARS-CoV-2, severe acute respiratory syndrome coronavirus 2.

[a] Three-level Multilevel regression analysis is an alternative way to control for geographic (county) and facility effect. We could not conduct multilevel competing risk models due to computational limitations. Therefore, we conducted three-level multilevel logistic regression models. Results from these models were similar to the main analysis that used conditional competing risk models conditioned on nursing homes, suggesting that both approaches adequately controlled for geographic and facility effect and gave similar findings on the association of patient characteristics with 30-day hospitalization and mortality.

[b] Functional impairment was categorized as no dependence (ADL score 0-8), mild (ADL score 9-16), moderate (ADL score 17-24) and severe dependence (ADL score 25-32).

[c] Use of catheter/tube included indwelling catheter, parenteral IV and feeding tube.

[d] Heart disease included Coronary artery disease, heart failure and hypertension.

[e] Neurologic conditions included stroke, hemiplegia and paraplegia.

[f] Respiratory conditions included chronic obstructive pulmonary disease, respiratory failure and shortness of breath.

© 2021 Mehta HB et al. *JAMA Network Open.*
